# Engineering Plant Immunity: Using CRISPR/Cas9 to Generate Virus Resistance

**DOI:** 10.3389/fpls.2016.01673

**Published:** 2016-11-08

**Authors:** Syed Shan-e-Ali Zaidi, Manal Tashkandi, Shahid Mansoor, Magdy M. Mahfouz

**Affiliations:** ^1^Laboratory for Genome Engineering, Division of Biological Sciences, King Abdullah University of Science and TechnologyThuwal, Saudi Arabia; ^2^National Institute for Biotechnology and Genetic EngineeringFaisalabad, Pakistan

**Keywords:** plant virus, CRISPR/Cas9, genome engineering, geminivirus, virus resistance

## Abstract

Plant viruses infect many economically important crops, including wheat, cotton, maize, cassava, and other vegetables. These viruses pose a serious threat to agriculture worldwide, as decreases in cropland area *per capita* may cause production to fall short of that required to feed the increasing world population. Under these circumstances, conventional strategies can fail to control rapidly evolving and emerging plant viruses. Genome-engineering strategies have recently emerged as promising tools to introduce desirable traits in many eukaryotic species, including plants. Among these genome engineering technologies, the CRISPR (clustered regularly interspaced palindromic repeats)/CRISPR-associated 9 (CRISPR/Cas9) system has received special interest because of its simplicity, efficiency, and reproducibility. Recent studies have used CRISPR/Cas9 to engineer virus resistance in plants, either by directly targeting and cleaving the viral genome, or by modifying the host plant genome to introduce viral immunity. Here, we briefly describe the biology of the CRISPR/Cas9 system and plant viruses, and how different genome engineering technologies have been used to target these viruses. We further describe the main findings from recent studies of CRISPR/Cas9-mediated viral interference and discuss how these findings can be applied to improve global agriculture. We conclude by pinpointing the gaps in our knowledge and the outstanding questions regarding CRISPR/Cas9-mediated viral immunity.

## Introduction

In the context of the rapidly growing global population, food security has emerged as one of the major challenges facing our generation (Cheeseman, 2016). The global population has increased by 60%, but *per capita* production of grains has fallen worldwide in the last 20 years (Suweis et al., 2015). If the population growth rate, which is 1.13 percent per year for 2016^1^ persists, the world population will double again within a mere 50 years, and it is estimated that food production will need to at least double till 2050 to meet demand (Suweis et al., 2015). Increases in food production per unit of land have not kept pace with increases in population and cropland area *per capita* has fallen by more than half since 1960 (Cheeseman, 2016).

### Plant Viruses

Agriculture worldwide is threatened by abiotic (heat, drought, frost, salinity, etc.) and biotic stresses (insect pests, fungi, bacteria, viruses, etc.). Among biotic stresses, phytopathogenic viruses cause an estimated 10–15% reduction in global crop yields each year (Mahy and van Regenmortel, 2009). Thus, improving host plant resistance against plant viruses can mitigate these losses by protecting a significant proportion of food crops.

The mechanisms of virus infection and transmission give many potential targets for controlling viruses in crop plants; however, the diversity of viruses and their rapid evolution make such approaches difficult (Hanley-Bowdoin et al., 2013). Most plant viruses are transmitted from one plant to another by a vector, an organism that feeds on the plant and transmits the virus from one plant to another (Hogenhout et al., 2008). The major vectors of plant viruses are insects (whiteflies, hoppers, thrips, beetles, etc.), mites, nematodes, and plasmodiophorids (Whitfield et al., 2015). Virus-infected plants show a range of symptoms depending on the pathogen; these symptoms often include leaf yellowing, leaf distortion, leaf curling, and other growth distortions like stunting of the whole plant, abnormalities in flower or fruit formation, etc. (Ghoshal and Sanfacon, 2015). Plant viruses are classified into six major groups based on their genomes: double-stranded DNA (dsDNA) viruses, single-stranded DNA (ssDNA) viruses, reverse-transcribing viruses, double-stranded RNA (dsRNA) viruses, negative sense single-stranded RNA (ssRNA-) viruses, and positive sense single-stranded RNA (ssRNA+) viruses (Roossinck, 2011; Roossinck et al., 2015). Most of the work on CRISPR-mediated viral interference has been done on the ssDNA geminiviruses; therefore, in this review, we focus on geminiviruses.

### Geminiviruses

Plant viruses belonging to family *Geminiviridae* infect important crops of several families including Cucurbitaceae (gourds, squash, watermelon, and melon), Euphorbiaceae (cassava), Solanaceae (tobacco, petunia, pepper, tomato, and potato), Malvaceae (okra, cotton), and Fabaceae (cowpea, mung bean, common bean, lima bean, and soybean) in different regions of the world (Seal et al., 2006; Zaidi et al., 2016a,c). Geminiviruses are characterized by their quasi-icosahedral twinned particles, which are approximately 18 × 30 nm in size and encapsidate circular, ssDNA of ∼2.5–3.1 kb (Stanley, 1985).

Based upon their host ranges, insect vectors and genome organizations, geminiviruses are classified into seven genera: *Begomovirus*, *Curtovirus*, *Topocuvirus*, *Mastrevirus*, *Becurtovirus*, *Turncurtovirus*, and *Eragrovirus* (Varsani et al., 2014; Brown et al., 2015). Most of the economically important geminiviruses are members of the genus *Begomovirus*. Begomoviruses are transmitted by the sweet potato/tobacco/silverleaf whitefly, *Bemisia tabaci* (Gennadius) (Order: Hemiptera, Family: Aleyrodidae), in a circulative persistent manner and are mostly restricted to the phloem of infected plants (Gilbertson et al., 2015).

**FIGURE 1:**
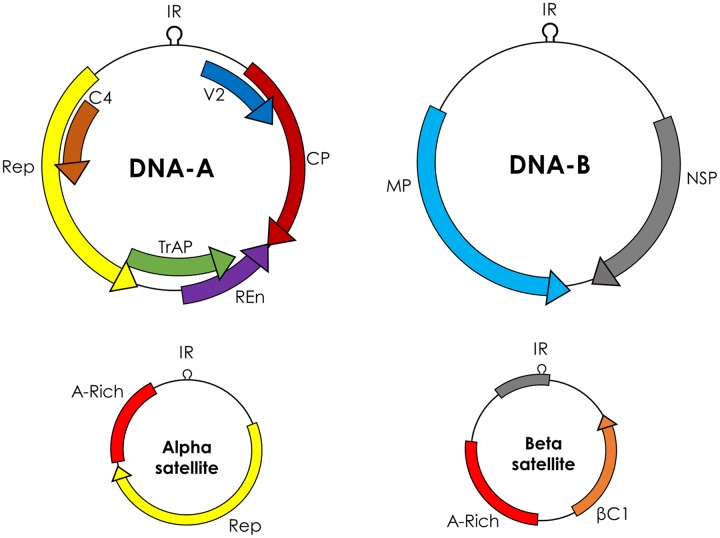
**Genome organization of begomoviruses and their associated alphasatellites and betasatellites.** Monopartite begomoviruses contain only a DNA-A like genome with genes for coat protein (CP) and V2 in sense orientation and replication associated protein (Rep), replication enhancer protein (REn), transcriptional activator protein (TrAP), and C4 in complementary sense orientation. Bipartite begomoviruses contain an extra genomic component DNA-B with genes for nuclear shuttle protein (NSP) in virion sense orientation and movement protein (MP) in complementary sense orientation. Old World (OW) begomoviruses are mostly associated with DNA satellites, which are half the size of the virus, called alphasatellites, and betasatellites. Alphasatellites encode Rep in virion sense orientation and betasatellites encode betaC1 in complementary sense orientation. Both satellites have adenine-rich regions (A-rich). The most conserved region among all begomoviruses and betasatellites is a nonanucleotide (TAATATT/AC) within the intergenic region (IR).

*Begomovirus* includes 288 species^2^ classified in two groups, based on their genome organization: monopartite (which have a single genome component), and bipartite (two genome components, DNA-A and DNA-B). DNA-A and DNA-B are 2.7–2.8 kb and each component has its own ORFs in a bidirectional fashion. Monopartite begomoviruses (or DNA-A of bipartite begomoviruses) have six ORFs, four in complementary sense orientation (*AC1/C1*–*AC4/C4*) and two in virion sense orientation (*AV1/V1* and *AV2/V2*; **Figure 1**). The ORF *AV2* is missing in begomoviruses from the New World. All proteins encoded by begomoviruses are multifunctional and are given names according to their functions. *AC1/C1* encodes replication-associated protein (Rep), *AC2/C2* encodes replication enhancer protein (REn), *AC3/C3* encodes transcriptional activator protein (TrAP), and *AC4/C4* encodes AC4/C4 protein. The ORF *AV1/V1* encodes for the coat protein (CP) while *AV2/V2* encodes another protein called pre-coat protein. The DNA-B component of bipartite begomoviruses encodes nuclear shuttle protein (NSP) from the *BC1* ORF and movement protein (MP) from the *BV1* ORF (Fondong, 2013).

The genes in the virion and complementary sense orientations on DNA-A and DNA-B are separated by an intergenic region (IR) containing a common region (CR) of sequences that are conserved between DNA-A and DNA-B. The main topological feature of the CR is a hairpin structure with a conserved nonanucleotide (TAATATT/AC) that spans the virion strand origin of replication (*v-ori*, indicated by the “/”) (Padidam et al., 1996). Iterated ∼5–7 nt long sequences (called iterons) that are present 5′ of the hairpin form binding sites for the virus replication-associated protein, Rep (encoded by *AC1*) (Chatterji et al., 2000). Begomoviruses in the Old World are mostly associated with symptom/pathogenicity determinant betasatellites and self-replicating alphasatellites (Zhou, 2013). Betasatellites encode βC1 protein and play critical role in important diseases like cotton leaf curl disease in the Indian Subcontinent (Briddon et al., 2014).

### Virus Control Strategies

Conventional virus control strategies focus on vector management using pesticides, activating natural predators, or the use of physical barriers like reflective mulches and UV-absorbing sheets (Legg et al., 2014). Additionally, culture practices like early sowing, weed management, crop-free periods, virus-free planting material, and the removal of infected plants have also been adopted for disease control. However, the complex epidemiological factors associated with virus disease outbreaks, such as vector migration dynamics, rapid virus evolution, and unpredictable virus host-range expansions, have all made it very difficult to develop effective long-term disease management strategies (Loebenstein and Katis, 2014).

Utilizing genetic resistance in crop plants by boosting the plant cellular immunity against viruses is rationally the most effective strategy, since above mentioned conventional strategies are expensive, labor intensive and often ineffective, specifically in case of viral diseases (Whitham and Hajimorad, 2016). The most effective way of achieving this goal will likely be the development of plant genotypes that are resistant/immune to the virus and/or vector, and using these in combination with other control measures. Therefore, a continuous research is going on to understand the plant cellular mechanisms for virus and virus vector resistance (Mandadi and Scholthof, 2013). Several such mechanisms have been discovered and artificially introduced or enhanced within plants to successfully demonstrate engineered virus resistance (Sahu and Prasad, 2015).

### Genome Engineering

Genome engineering has recently emerged as a ground breaking tool to improve several eukaryotic species, including crop plants, by introducing several traits of interest through the site-specific modification of the genome (Sovova et al., 2016). In addition to its ease and reproducibility, one attractive feature of these technologies is that once the desired genome alterations have been made, the transgenes can be crossed out from the improved variety, thus circumventing public and political concerns around the use of persistent transgenes in crops (Woo et al., 2015; Kanchiswamy, 2016; Zhang et al., 2016).

**FIGURE 2:**
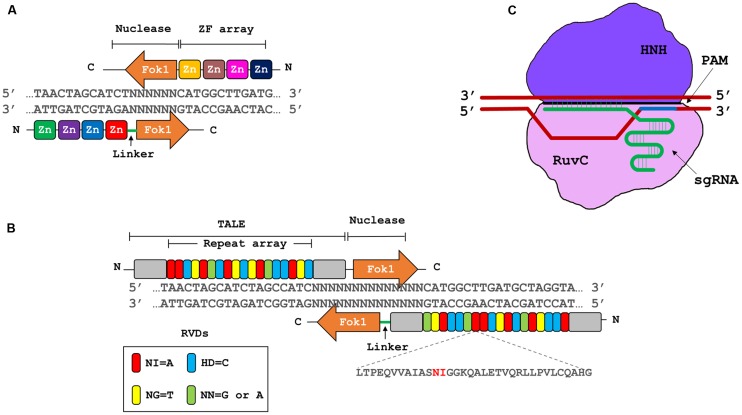
**Three major types of genome engineering platforms. (A)** Zinc finger nucleases (ZFNs). ZFNs contains artificial zinc finger motifs’ array, that dictate the iteration of a nucleotide triplet; and a type II restriction endonuclease FokI, that produces double stranded breaks (DSBs). **(B)** Transcription activator-like effector nucleases (TALENs). TALENs are based on type III secretory systems of *Xanthomonas* sp. The DNA binding domain of TALE array consists of highly conserved 33–34 residue long repetitive motifs, containing repeat variable di-residues (RVDs) at positions 12 and 13 to dictate the specific binding site. The nuclease domains contain FokI endonuclease to produce DSBs. **(C)** Clustered regularly interspaced short palindromic repeat/CRISPR associated9 (CRISPR/Cas9) system. This system consists of a single guide RNA (sgRNA) and Cas9 endonuclease from *Streptococcus pyogenes*, with its two domains, RuvC and HNH. sgRNA defines the specific site to be targeted where Cas9 nuclease produces DSBs 3 base pairs upstream of protospacer adjacent motif (PAM, NGG in the case of *S. pyogenes*).

Genome engineering refers to the use of site specific nucleases (SSNs), that can be designed to bind and cleave a specific nucleic acid sequence by introducing double stranded breaks (DSBs) at or near the target site (Piatek and Mahfouz, 2016). There are four major classes of SSNs: meganucleases, zinc finger nucleases (ZFNs), transcription activator-like effector nucleases (TALENs), and clustered regularly interspaced palindromic repeats/CRISPR-associated 9 (Stella and Montoya, 2016) (CRISPR/Cas9; **Figure 2**). These techniques have been harnessed independently to improve crop plant resistance to viruses directly by targeting viral genomes or by targeting host factors (**Table 1**).

**Table 1:** Sequence-specific nucleases designed to engineer virus resistance.

Nuclease	Virus	Plant	Target	References
Zinc finger nuclease (ZFN)	TYLCCNV and TbCSV	*Nicotiana benthamiana*	Rep	Chen et al., 2014
	TYLCV	*Arabidopsis thaliana*	Rep binding site in IR	Mori et al., 2013
	TYLCV	*A. thaliana*	Rep binding site in IR	Koshino-Kimura et al., 2009
	TYLCV	*A. thaliana*	Rep binding site in IR	Koshino-Kimura et al., 2008
	TYLCV	*A. thaliana*	Rep binding site in IR	Takenaka et al., 2007
	BSCTV	*A. thaliana*	Rep binding site in IR	Sera, 2005
Transcription activator-like effector nuclease (TALEN)	TbCSV, TLCYnV, TYLCCNV, and TYLCCNB	*N. benthamiana*	Rep and Hairpin in IR	Cheng et al., 2015
Clustered regularly interspaced palindromic repeats/CRISPR-associated 9 (CRISPR/Cas9)	TYLCV, BCTV, and MeMV BeYDV BSCTV CLCuKoV, TYLCV 2.3, TYLCSV, MeMV, BCTV-Logan, BCTV-Worland TuMV CVYV, ZYMV, and PRSMV	*N. benthamianaN. benthamiana* *N. benthamiana* and *A. thaliana* *N. benthamiana* *A. thaliana* *Cucumis sativus*	IR, CP, and Rep LIR and Rep/RepA IR, CP, and Rep IR, CP, and Rep Host factor *eIF(iso)4E* Host factor *eIF4E*	Ali et al., 2015a Baltes et al., 2015 Ji et al., 2015 Ali et al., 2016 Pyott et al., 2016 Chandrasekaran et al., 2016

*Tomato yellow leaf curl China virus (TYLCCNV), Tobacco curly shoot virus (TbCSV), Tomato yellow leaf curl virus (TYLCV), Beet severe curly top virus (BSCTV), Tomato leaf curl Yunnan virus (TLCYnV), Tomato yellow leaf curl China betasatellite (TYLCCNB), Beet curly top virus (BCTV), Merremia mosaic virus (MeMV), Bean yellow dwarf virus (BeYDV), Cotton leaf curl Kokhran virus (CLCuKoV), Tomato yellow leaf curl Sardinian virus (TYLCSV), Turnip mosaic virus (TuMV), Cucumber vein yellowing virus (CVYV), Zucchini yellow mosaic virus (ZYMV), Papaya ring spot mosaic virus (PRSMV), replication associated protein (Rep), intergenic region (IR), long intergenic region (LIR), coat protein (CP).*

The DSBs generated by the SSNs can be repaired by one of two endogenous mechanisms: non-homologous end joining (NHEJ) or homologous recombination (Aouida et al., 2014, 2015a,b; Ali et al., 2015b; Piatek and Mahfouz, 2016). The most straightforward application of SSNs is to create gene knockouts through NHEJ (Barakate and Stephens, 2016). The repair of DSBs via NHEJ often leads to the formation of small insertion/deletion (indel) mutations (Wright et al., 2016). These indel mutations can disrupt coding or regulatory sequences of the target gene resulting in loss-of-function mutations. Repair of double-strand breaks by homologous recombination is more complex, because it requires the simultaneous delivery of a DNA repair template that carries the desired modification to be incorporated into the repaired locus (Stella and Montoya, 2016). Homologous recombination can be used for a variety of purposes like site specific gene insertion, stacking of genes at a specific genome position and genome alteration to a single base level (Nishida et al., 2016).

Here we discuss the utilization of different genome engineering platforms including ZFNs, TALENs, and CRISPR/Cas9 to engineer virus resistance in plants with respect to the technological advancements, limitations, and future prospects.

## Genome Engineering Strategies To Confer Virus Resistance

Initial studies to target plant viruses using genome engineering focused on ZFN to target Rep binding sites/iterons of begomoviruses. ZFN developed for iterons of *Beet severe curly top virus* (BSCTV) or *Tomato yellow leaf curl virus* (TYLCV) effectively targeted BSCTV and TYLCV, respectively (Sera, 2005; Mori et al., 2013). Transgenic *Arabidopsis thaliana* plants developed to target iteron of BSCTV demonstrated complete resistance (Sera, 2005). However, the work of Chen et al. (2014) supported that ZFN can efficiently target BSCTV and TYLCV but argued that this virus sequence specific strategy may not be effective in field where mixed virus infections are common. Alternately, targeting the conserved regions of virus would confer comparatively durable resistance. Three conserved regions among Rep of monopartite begomoviruses were identified and tested for broad-spectrum resistance. Among these three targets, one target of 25 base pairs effectively worked against *Tomato yellow leaf curl China viru*s (TYLCCNV) and *Tobacco curly shoot virus* (TbCSV) (Chen et al., 2014).

TALENs have been used as platform for designing broad-spectrum resistance to begomoviruses (Cheng et al., 2015). Two highly conserved targets, *AC1* and nonanucleotide, were selected and targeted using TALE. DNA binding efficiencies of TALE were confirmed and transgenic *Nicotiana benthamiana* plants were developed. Two begomoviruses alone, i.e., TbCSV and *Tomato leaf curl Yunnan virus* (TLCYnV); and one begomovirus with associated betasatellite, i.e., TYLCCNV with its cognate Tomato yellow leaf curl China betasatellite (TYLCCNB), were tested. Transgenic plants demonstrated partial resistance by developing delayed symptoms and reduced viral DNA accumulation (Cheng et al., 2015).

ZFNs and TALENs are effective genome engineering technologies but their major limitation is that tailoring the DNA binding proteins to target a sequence of interest can be costly and time-consuming (Ceasar et al., 2016). Furthermore, engineering TALENs to generate targeted DSBs requires two TALEN proteins capable of binding in a tail-to-tail orientation to facilitate the dimerization of FokI nuclease domain (Sun and Zhao, 2013). These, and other, limitations were considerably reduced in the past few years due to the advent, development, and subsequent technological advancements of the CRISPR/Cas9 system (Stella and Montoya, 2016).

## Engineering Crispr/Cas9-Based Resistance Against Dna Viruses

CRISPR/Cas9 is a prokaryotic molecular immunity system against invading nucleic acids (through horizontal gene transfer) and phages (Marraffini and Sontheimer, 2008). Bacteria and archaea acquire short pieces, or spacers, from these invading nucleic acids and incorporate them within their genomes, where they serve as molecular memory (Bolotin et al., 2005). During subsequent infections, these short pieces are transcribed as part of the CRISPR array; after transcription and maturation, CRISPR RNA (crRNA) can help guide the Cas9 endonuclease to scan the invading DNA and cleave the target sequence (Nunez et al., 2016). The Cas9 endonuclease cleaves the target sequence at a site preceding the protospacer-associated motif (PAM), which is NGG for *Streptomyces pyogenes* Cas9 (Wright et al., 2016) (**Figure 2C**). CRISPR/Cas9 is one of the most widely adapted systems for genome engineering and has been used successfully in several species ranging from simple microbes to complex plants and animals (Hsu et al., 2014).

Several mammalian infecting DNA viruses have been targeted and mutagenized using SSNs (Schiffer et al., 2012), for example the CRISPR/Cas9 system was used to engineer host genome and confer resistance against human immunodeficiency virus (HIV) infection (Hu et al., 2014). Other viruses like herpes simplex virus (Suenaga et al., 2014), latent infection by Epstein-Barr virus (Wang and Quake, 2014; Yuen et al., 2015) and hepatitis B virus (Zhen et al., 2015) have also been targeted using CRISPR/Cas9. Moreover, targeting of RNA viruses using similar approach have also been demonstrated (Price et al., 2015).

**FIGURE 3:**
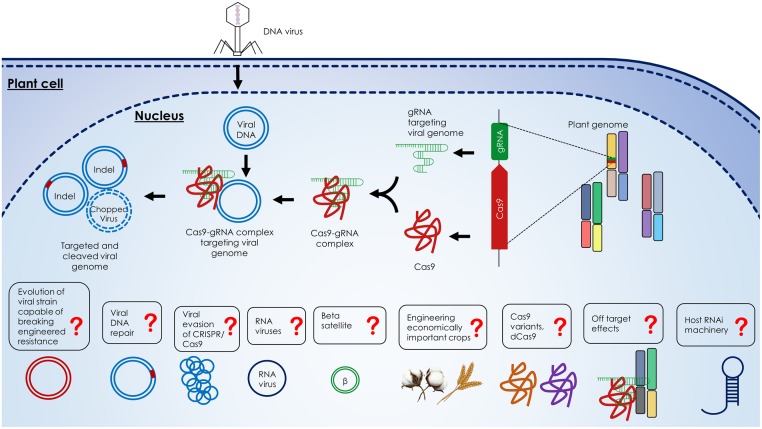
**Description of the CRISPR/Cas9-mediated virus interference in the plant cell.** Components of the CRISPR/Cas9 machinery, gRNA, and Cas9, are expressed from the plant genome and form gRNA-Cas9 complex. Upon viral infection, the viral DNA replicates through the dsDNA replicative form inside the nucleus of host cell. The gRNA-Cas9 complex targets the viral dsDNA at complementary target sites and cleaves the viral genome via double strand breaks (DSBs) formation which can be repaired by non-homologous end joining (NHEJ) repair. Alternatively, the formation of DSBs can lead to the degradation of the virus genome. The outstanding questions like the response of host RNAi machinery to the CRISPR/Cas9 system, off target effects of CRISPR/Cas9 system on host genome, the use of catalytically inactive Cas9 variants (dCas9), engineering of economically important crops, targeting betasatellites and RNA viruses with the CRISPR/Cas9 system, evasion of virus from the CRISPR/Cas9 system, host proteins involved in viral DNA repair and evolution of resistance breaking viral strains are highlighted by question marks.

Four recent studies demonstrated the power of the CRISPR/Cas9 system to efficiently confer resistance to geminiviruses in plants (**Figure 3**) (Ali et al., 2015a, 2016; Baltes et al., 2015; Ji et al., 2015). Ali et al. (2015a) showed that *N. benthamiana* plants expressing the CRISPR/Cas9 machinery exhibited resistance against TYLCV, *Beet curly top virus* (BCTV), and *Merremia mosaic virus* (MeMV). Baltes et al. (2015) and Ji et al. (2015) demonstrated virus interference activities in *N. benthamiana* against *Bean yellow dwarf virus* (BeYDV) and BSCTV, respectively. Ji et al. (2015) correlated Cas9 expression with the levels of virus suppression, indicating the need to use one background transgenic line with optimum expression of Cas9 and the sgRNA for practical applications. Baltes et al. (2015) showed that one sgRNA targeting the BeYDV genome could confer plant resistance without cleavage activity, which suggests that catalytically inactive Cas9 (dCas9) can be used to mediate virus interference, thereby eliminating concerns of off-target activities in the plant genome. In a follow-up study, Ali et al. (2016) demonstrated that this technology can be used to target and cleave *Cotton leaf curl Kokhran virus* (CLCuKoV) and also showed that targeting the conserved nonanucleotide sequence can target multiple begomoviruses simultaneously (CLCuKoV, TYLCV, TYLCSV, MeMV, BCTV-Worland, and BCTV-Logan), conferring broad-spectrum geminivirus resistance. All of these studies showed that *N. benthamiana* plants expressing the CRISPR/Cas9 system displayed considerably reduced viral titers, which abolished or significantly reduced disease symptoms.

Since there is an arms race between the invading viruses and their host plants Ali et al. (2016) systematically analyzed the ability of geminiviruses to evade the CRISPR/Cas9 machinery. They determined the ability of multiple geminiviruses to evade the CRISPR/Cas9 machinery by targeting coding and non-coding sequences. This study reveals that targeting coding sequences led to the generation of viral variants capable of evading the CRISPR/Cas9 machinery. Interestingly, targeting the non-coding intergenic sequences led to high levels of virus interference, no detectable viral escapes from the CRISPR/Cas9 machinery, and thereby providing an effective strategy to confer potential durable resistance.

## Engineering Crispr/Cas9-Based Resistance Against Rna Viruses

Currently there is no report of directly targeting and cleaving RNA viruses. The limitation of directly targeting RNA viral genomes is that the guide RNA-Cas9 system can only be used to target DNA viruses. This could change in the future, because Cas9 can be programmed to cleave RNA (O’Connell et al., 2014) and the Type III-B CRISPR-Cas system mediates programmable cleavage of RNA sequences that are complementary to a guide RNA (Hale et al., 2009). These Cas9 variants have the potential to target and cleave RNA viruses within plant cells. However, whether this targeting can work as efficiently for RNA viruses as it has for DNA viruses, remains to be explored.

Rather than targeting the virus genome, genome-editing strategies to create resistance to RNA viruses might target the plant genome. In this approach, the CRISPR/Cas9 system can modify plant genes that affect plant responses to viral infection and thus generate virus resistance.

Translation initiation like factors, *eIF4E* and *eIF(iso)4E*, have been demonstrated to be directly involved in the infection of RNA viruses (Sanfacon, 2015). *A. thaliana* mutant plants of these translation initiation factors exhibited resistance to *Turnip mosaic virus* (TuMV) (Lellis et al., 2002). Therefore these targets were mutated with the CRISPR/Cas9 system, in order to develop virus resistant plants (Chandrasekaran et al., 2016; Pyott et al., 2016). The utility of CRISPR/Cas9 technology for generating novel genetic resistance to the potyvirus TuMV was demonstrated in *A. thaliana* by deletion of a host factor, *eIF(iso)4E*, which is strictly required for viral survival (Pyott et al., 2016). Development of virus resistance in cucumber (*Cucumis sativus* L.) was also demonstrated by disrupting *eIF4E*, and developing non-transgenic heterozygous *eIF4E* mutant plants. These non-transgenic plants developed partial resistance to an ipomovirus (*Cucumber vein yellowing virus*) and two potyviruses (*Zucchini yellow mosaic virus* and *Papaya ring spot mosaic virus*-W) (Chandrasekaran et al., 2016).

The translation initiation factors are prime candidates for host genes that can be targeted (Sanfacon, 2015), but any host gene encoding a factor that the virus requires is a potential target for modification. This form of recessive resistance could be exploited with the aid of the CRISPR/Cas9 system to create novel resistance alleles in crop plants to protect them against problematic viruses that use host translation initiation factors.

## Applications

The CRISPR/Cas9 system can be used to engineer, so-called ‘non-transgenic’ virus-resistant varieties (Woo et al., 2015). A major advantage of targeting or modifying host factors is that CRISPR/Cas9 can be introduced as transgenes to create the genome edits, and then progeny plants can be selected that carry the desired edits but have lost the Cas9 transgene through segregation (Kanchiswamy, 2016). Alternatively, the Cas9 protein and other reagents like the guide RNA may be introduced as ribonucleoprotein complex (RNP) directly into cells, which would not involve the incorporation of transgenes into the genome. The resulting plants would therefore be indistinguishable from plants carrying naturally occurring alleles or plants identified from screens following random mutagenesis (Voytas and Gao, 2014). These approaches are applicable to viruses with RNA or DNA genomes. Targeting host factors like *eIF4E* and *eIF(iso)4E* have certain additional advantages; for example, many natural sources of *Potyvirus* resistance rely on loss-of-function mutations in host *eIF*s (Sanfacon, 2015). Therefore, loss-of-function mutations in *eIF*s should theoretically provide broad-spectrum resistance. Moreover, the commercial application of such a strategy might bypass some biosafety regulations on genetically modified organisms, as the final genome-edited product is essentially no different from varieties carrying mutant alleles that arose from ‘natural’ methods of mutagenesis. CRISPR-edited non-transgenic mushrooms and maize, developed using a similar strategy, are on their way to being commercialized in the US (Waltz, 2016a,b). It is noteworthy that in some applications, engineered viral resistance results from a single nucleotide point mutation produced by the plant’s own natural DNA damage repair mechanism, namely NHEJ.

The CRISPR/Cas9 system has mostly been used to engineer immunity in model plants. Several viral diseases that cause devastating losses of many economically important crops around the world still must be addressed. Important diseases that remain a challenge include cassava mosaic disease, a severe and widespread disease that limits cassava production in sub-Saharan Africa (Legg, 2008); tomato yellow leaf curl disease, which causes heavy damage to tomato crops in the Mediterranean area, Central America, and Asia (Czosnek, 2008); and cotton leaf curl disease, the major limiting factor for cotton production in the Indian subcontinent (Mansoor et al., 2008; Zaidi et al., 2016d). The successful demonstration that CRISPR/Cas9 can be used to confer resistance against viruses in model plants indicates the potential of this technique to control these important viral diseases in key economic crops.

In addition to engineering virus resistance, CRISPR/Cas9 can be used to address several basic biological questions. Virus replication within the host cell has been thoroughly studied (Hanley-Bowdoin et al., 2013), but viral DNA repair remains poorly understood. Moreover, the rate of viral DNA recombination and mutation, after the viral genome is cleaved within the host cell, also remains to be explored. Other outstanding questions include whether multiplexing gRNAs in transgenic plants can facilitate resistance to multiple viruses, as occurs naturally in crRNA in prokaryotic CRISPR arrays (Wright et al., 2016). The existence of off-target effects when CRISPR/Cas9 is used *in vivo* in actual hosts also remains to be explored. The efficacy of engineered viral immunity in economically important crops, like wheat and cotton; and the targeting of pathogenicity determinant betasatellites, key player in several important begomoviral diseases, also remain to be explored. How does the plant defense system respond to the foreign CRISPR/Cas9 system? Will the host’s RNA interference machinery interfere with CRISPR/Cas9 function? How can viruses evade CRISPR/Cas9-mediated interference? (**Figure 3**)

## Future Perspective And Conclusion

The advent of CRISPR/Cas9 technology has revolutionized the field of genome editing. Crucially, the fact that the Cas9 nuclease is guided by RNA rather than protein overcomes the major limitations of TALEN and ZFN technologies. RNA-based guiding is cheaper and easier to engineer and greatly expands the range of possible target sequences, requiring only the commonly occurring NGG PAM sequence and other variants.

Although the efficacy of genome engineering techniques has been demonstrated for production of resistance to plant viruses in several studies (Zaidi et al., 2016b), it remains to be determined whether these techniques are effective under natural conditions in open field trials. Geminiviruses have already been shown to evade CRISPR/Cas9-mediated resistance when viral coding regions are targeted (Ali et al., 2016). Whether CRISPR/Cas9 could accelerate geminivirus evolution remains an important question. Targeted cleavage of a geminivirus could expedite viral evasion of the CRISPR/Cas9 system, or stimulate accelerated evolution of viral strains that can evade the CRISPR machinery.

Notably, the durability of this engineered resistance also remains to be tested. Recessive resistance arising from the loss of a host factor required by the virus is assumed to be more durable than dominant *R* genes, due to lower selective pressures on the virus to evolve counter defense strategies (de Ronde et al., 2014). The resistance breaking of CRISPR/Cas9-induced recessive mutants also remains to be tested. The coming years will provide more detailed analyses of these technologies, and will eventually lead to their use in development of a variety of marketable crops.

## Author Contributions

MM and SM provided the outlines of the review and contributed the key ideas. SZ and MT wrote the manuscript and prepared the figures. MM, SZ, MT, and SM worked on and improved the original draft and figures.

## Conflict of Interest Statement

The authors declare that the research was conducted in the absence of any commercial or financial relationships that could be construed as a potential conflict of interest.

## Footnotes

1 www.worldometers.info/world-population

2 www.ictvonline.org

## References

[B1] Ali Z. Abulfaraj A. Idris A. Ali S. Tashkandi M. Mahfouz M. M. (2015a). CRISPR/Cas9-mediated viral interference in plants. *Genome Biol.* 16:238 10.1186/s13059-015-0799-6 PMC4641396 26556628

[B2] Ali Z. Abul-Faraj A. Piatek M. Mahfouz M. M. (2015b). Activity and specificity of TRV-mediated gene editing in plants. *Plant Signal. Behav.* 10:e1044191 10.1080/15592324.2015.1044191 PMC4883890 26039254

[B3] Ali Z. Ali S. Tashkandi M. Zaidi S. S. Mahfouz M. M. (2016). CRISPR/Cas9-mediated immunity to geminiviruses: differential interference and evasion. *Sci. Rep.* 6:26912 10.1038/srep30223 PMC4881029 27225592

[B4] Aouida M. Eid A. Ali Z. Cradick T. Lee C. Deshmukh H. (2015a). Efficient fdCas9 synthetic endonuclease with improved specificity for precise genome engineering. *PLoS ONE* 10:e0133373 10.1371/journal.pone.0133373 PMC4520497 26225561

[B5] Aouida M. Li L. Mahjoub A. Alshareef S. Ali Z. Piatek A. (2015b). Transcription activator-like effector nucleases mediated metabolic engineering for enhanced fatty acids production in *Saccharomyces cerevisiae*. *J. Biosci. Bioeng.* 120 364–371. 10.1016/j.jbiosc.2015.02.017 25907574

[B6] Aouida M. Piatek M. J. Bangarusamy D. K. Mahfouz M. M. (2014). Activities and specificities of homodimeric TALENs in *Saccharomyces cerevisiae*. *Curr. Genet.* 60 61–74. 10.1007/s00294-013-0412-z 24081604

[B7] Baltes N. J. Hummel A. W. Konecna E. Cegan R. Bruns A. N. Bisaro D. M. (2015). Conferring resistance to geminiviruses with the CRISPR–Cas prokaryotic immune system. *Nat. Plants* 1:15145 10.1038/nplants.2015.145 PMC8612103 34824864

[B8] Barakate A. Stephens J. (2016). An overview of CRISPR-based tools and their improvements: new opportunities in understanding plant-pathogen interactions for better crop protection. *Front. Plant Sci.* 7:765 10.3389/fpls.2016.00765 PMC4887484 27313592

[B9] Bolotin A. Quinquis B. Sorokin A. Ehrlich S. D. (2005). Clustered regularly interspaced short palindrome repeats (CRISPRs) have spacers of extrachromosomal origin. *Microbiology* 151 2551–2561. 10.1099/mic.0.28048-0 16079334

[B10] Briddon R. W. Akbar F. Iqbal Z. Amrao L. Amin I. Saeed M. (2014). Effects of genetic changes to the begomovirus/betasatellite complex causing cotton leaf curl disease in South Asia post-resistance breaking. *Virus Res.* 186 114–119. 10.1016/j.virusres.2013.12.008 24361351

[B11] Brown J. K. Zerbini F. M. Navas-Castillo J. Moriones E. Ramos-Sobrinho R. Silva J. C. (2015). Revision of *Begomovirus* taxonomy based on pairwise sequence comparisons. *Arch. Virol.* 160 1593–1619. 10.1007/s00705-015-2398-y 25894478

[B12] Ceasar S. A. Rajan V. Prykhozhij S. V. Berman J. N. Ignacimuthu S. (2016). Insert, remove or replace: a highly advanced genome editing system using CRISPR/Cas9. *BBA-Mol. Cell Res.* 1863 2333–2344. 10.1016/j.bbamcr.2016.06.009 27350235

[B13] Chandrasekaran J. Brumin M. Wolf D. Leibman D. Klap C. Pearlsman M. (2016). Development of broad virus resistance in non-transgenic cucumber using CRISPR/Cas9 technology. *Mol. Plant Pathol.* 17 1140–1153. 10.1111/mpp.12375 26808139 PMC6638350

[B14] Chatterji A. Chatterji U. Beachy R. N. Fauquet C. M. (2000). Sequence parameters that determine specificity of binding of the replication-associated protein to its cognate site in two strains of Tomato leaf curl virus-New Delhi. *Virology* 273 341–350. 10.1006/viro.2000.0434 10915605

[B15] Cheeseman J. (2016). “7 - food security in the face of salinity, drought, climate change, and population growth,” in *Halophytes for Food Security in Dry Lands*, ed. Uham M. (San Diego, CA: Academic Press), 111–123.

[B16] Chen W. Qian Y. Wu X. Sun Y. Wu X. Cheng X. (2014). Inhibiting replication of begomoviruses using artificial zinc finger nucleases that target viral-conserved nucleotide motif. *Virus Genes* 48 494–501. 10.1007/s11262-014-1041-4 24474330

[B17] Cheng X. Li F. Cai J. Chen W. Zhao N. Sun Y. (2015). Artificial TALE as a convenient protein platform for engineering broad-spectrum resistance to begomoviruses. *Viruses* 7 4772–4782. 10.3390/v7082843 26308041 PMC4576204

[B18] Czosnek H. (2008). “Tomato yellow leaf curl virus,” in *Encyclopedia of Virology*, eds Mahy B. W. J. Van Regenmortel M. H. V. (Oxford: Academic Press), 138–145.

[B19] de Ronde D. Butterbach P. Kormelink R. (2014). Dominant resistance against plant viruses. *Front. Plant Sci.* 5:307 10.3389/fpls.2014.00307 PMC4073217 25018765

[B20] Fondong V. N. (2013). Geminivirus protein structure and function. *Mol. Plant Pathol.* 14 635–649. 10.1111/mpp.12032 23615043 PMC6638828

[B21] Ghoshal B. Sanfacon H. (2015). Symptom recovery in virus-infected plants: revisiting the role of RNA silencing mechanisms. *Virology* 479–480, 167–179. 10.1016/j.virol.2015.01.008 25677651

[B22] Gilbertson R. L. Batuman O. Webster C. G. Adkins S. (2015). Role of the insect supervectors *Bemisia tabaci* and *Frankliniella occidentalis* in the emergence and global spread of plant viruses. *Annu. Rev. Virol.* 2 67–93. 10.1146/annurev-virology-031413-085410 26958907

[B23] Hale C. R. Zhao P. Olson S. Duff M. O. Graveley B. R. Wells L. (2009). RNA-guided RNA cleavage by a CRISPR RNA-Cas protein complex. *Cell* 139 945–956. 10.1016/j.cell.2009.07.040 19945378 PMC2951265

[B24] Hanley-Bowdoin L. Bejarano E. R. Robertson D. Mansoor S. (2013). Geminiviruses: masters at redirecting and reprogramming plant processes. *Nat. Rev. Microbiol.* 11 777–788. 10.1038/nrmicro3117 24100361

[B25] Hogenhout S. A. Ammar El D. Whitfield A. E. Redinbaugh M. G. (2008). Insect vector interactions with persistently transmitted viruses. *Annu. Rev. Phytopathol.* 46 327–359. 10.1146/annurev.phyto.022508.092135 18680428

[B26] Hsu P. D. Lander E. S. Zhang F. (2014). Development and applications of CRISPR-Cas9 for genome engineering. *Cell* 157 1262–1278. 10.1016/j.cell.2014.05.010 24906146 PMC4343198

[B27] Hu W. Kaminski R. Yang F. Zhang Y. Cosentino L. Li F. (2014). RNA-directed gene editing specifically eradicates latent and prevents new HIV-1 infection. *Proc. Natl. Acad. Sci. U.S.A.* 111 11461–11466. 10.1073/pnas.1405186111 25049410 PMC4128125

[B28] Ji X. Zhang H. Zhang Y. Wang Y. Gao C. (2015). Establishing a CRISPR-Cas-like immune system conferring DNA virus resistance in plants. *Nat. Plants* 1:15144 10.1038/nplants.2015.144 27251395

[B29] Kanchiswamy C. N. (2016). DNA-free genome editing methods for targeted crop improvement. *Plant Cell Rep.* 35 1469–1474. 10.1007/s00299-016-1982-2 27100964

[B30] Koshino-Kimura Y. Takenaka K. Domoto F. Aoyama Y. Sera T. (2008). Generation of plants resistant to tomato yellow leaf curl virus by using artificial zinc-finger proteins. *Nucleic Acids Symp. Ser. (Oxf.)* 52 189–190. 10.1093/nass/nrn096 18776317

[B31] Koshino-Kimura Y. Takenaka K. Domoto F. Ohashi M. Miyazaki T. Aoyama Y. (2009). Construction of plants resistant to TYLCV by using artificial zinc-finger proteins. *Nucleic Acids Symp. Ser. (Oxf.)* 53 281–282. 10.1093/nass/nrp141 19749370

[B32] Legg J. P. (2008). “African cassava mosaic disease,” in *Encyclopedia of Virology*, eds Mahy B. W. J. Van Regenmortel M. H. V. (Oxford: Academic Press), 30–36.

[B33] Legg J. P. Shirima R. Tajebe L. S. Guastella D. Boniface S. Jeremiah S. (2014). Biology and management of Bemisia whitefly vectors of cassava virus pandemics in Africa. *Pest. Manag. Sci.* 70 1446–1453. 10.1002/ps.3793 24706604

[B34] Lellis A. D. Kasschau K. D. Whitham S. A. Carrington J. C. (2002). Loss-of-susceptibility mutants of *Arabidopsis thaliana* reveal an essential role for eIF(iso)4E during potyvirus infection. *Curr. Biol.* 12 1046–1051. 10.1016/S0960-9822(02)00898-9 12123581

[B35] Loebenstein G. Katis N. (2014). “Control of plant virus diseases seed-propagated crops,” in *Advance Virus Reserch*, eds Gad L. Nikolaos K. (Cambridge, MA: Academic Press), 11. 10.1016/B978-0-12-801246-8.09985-6 25410109

[B36] Mahy B. W. J. van Regenmortel M. H. V. (2009). *Desk Encyclopedia of Plant and Fungal Virology.* Cambridge, MA: Academic Press.

[B37] Mandadi K. K. Scholthof K. B. (2013). Plant immune responses against viruses: how does a virus cause disease? *Plant Cell* 25 1489–1505. 10.1105/tpc.113.111658 23709626 PMC3694688

[B38] Mansoor S. Amin I. Briddon R. W. (2008). “Cotton leaf curl disease,” in *Encyclopedia of Virology*, eds Mahy B. W. J. Van Regenmortel M. H. V. (Oxford: Academic Press), 563–569.

[B39] Marraffini L. A. Sontheimer E. J. (2008). CRISPR interference limits horizontal gene transfer in staphylococci by targeting DNA. *Science* 322 1843–1845. 10.1126/science.1165771 19095942 PMC2695655

[B40] Mori T. Takenaka K. Domoto F. Aoyama Y. Sera T. (2013). Inhibition of binding of tomato yellow leaf curl virus rep to its replication origin by artificial zinc-finger protein. *Mol. Biotechnol.* 54 198–203. 10.1007/s12033-012-9552-5 22576255

[B41] Nishida K. Arazoe T. Yachie N. Banno S. Kakimoto M. Tabata M. (2016). Targeted nucleotide editing using hybrid prokaryotic and vertebrate adaptive immune systems. *Science* 353:aaf8729 10.1126/science.aaf8729 27492474

[B42] Nunez J. K. Harrington L. B. Doudna J. A. (2016). Chemical and biophysical modulation of Cas9 for tunable genome engineering. *ACS Chem. Biol.* 11 681–688. 10.1021/acschembio.5b01019 26857072

[B43] O’Connell M. R. Oakes B. L. Sternberg S. H. East-Seletsky A. Kaplan M. Doudna J. A. (2014). Programmable RNA recognition and cleavage by CRISPR/Cas9. *Nature* 516 263–266. 10.1038/nature13769 25274302 PMC4268322

[B44] Padidam M. Beachy R. N. Fauquet C. M. (1996). The role of AV2 (“precoat”) and coat protein in viral replication and movement in tomato leaf curl geminivirus. *Virology* 224 390–404. 10.1006/viro.1996.0546 8874500

[B45] Piatek A. Mahfouz M. M. (2016). Targeted genome regulation via synthetic programmable transcriptional regulators. *Crit. Rev. Biotechnol.* 10.3109/07388551.2016.1165180 [Epub ahead of print]. 27093352

[B46] Price A. A. Sampson T. R. Ratner H. K. Grakoui A. Weiss D. S. (2015). Cas9-mediated targeting of viral RNA in eukaryotic cells. *Proc. Natl. Acad. Sci. U.S.A.* 112 6164–6169. 10.1073/pnas.1422340112 25918406 PMC4434742

[B47] Pyott D. E. Sheehan E. Molnar A. (2016). Engineering of CRISPR/Cas9-mediated potyvirus resistance in transgene-free *Arabidopsis* plants. *Mol. Plant Pathol.* 17 1276–1288. 10.1111/mpp.12417 27103354 PMC5026172

[B48] Roossinck M. J. (2011). The big unknown: plant virus biodiversity. *Curr. Opin. Virol.* 1 63–67. 10.1016/j.coviro.2011.05.022 22440569

[B49] Roossinck M. J. Martin D. P. Roumagnac P. (2015). Plant virus metagenomics: advances in virus discovery. *Phytopathology* 105 716–727. 10.1094/PHYTO-12-14-0356-RVW 26056847

[B50] Sahu P. P. Prasad M. (2015). Application of molecular antiviral compounds: novel approach for durable resistance against geminiviruses. *Mol. Biol. Rep.* 42 1157–1162. 10.1007/s11033-015-3852-3 25652324

[B51] Sanfacon H. (2015). Plant translation factors and virus resistance. *Viruses* 7 3392–3419. 10.3390/v7072778 26114476 PMC4517107

[B52] Schiffer J. T. Aubert M. Weber N. D. Mintzer E. Stone D. Jerome K. R. (2012). Targeted DNA mutagenesis for the cure of chronic viral infections. *J. Virol.* 86 8920–8936. 10.1128/JVI.00052-12 22718830 PMC3416169

[B53] Seal S. E. Jeger M. J. Van Den Bosch F. (2006). Begomovirus evolution and disease management. *Adv. Virus Res.* 67 297–316. 10.1016/S0065-3527(06)67008-5 17027683

[B54] Sera T. (2005). Inhibition of virus DNA replication by artificial zinc finger proteins. *J. Virol.* 79 2614–2619. 10.1128/JVI.79.4.2614-2619.2005 15681461 PMC546585

[B55] Sovova T. Kerins G. Demnerova K. Ovesna J. (2016). Genome editing with engineered nucleases in economically important animals and plants: state of the art in the research pipeline. *Curr. Issues Mol. Biol.* 21 41–62. 27253613

[B56] Stanley J. (1985). The molecular biology of geminiviruses. *Adv. Virus Res.* 30 139–177. 10.1016/S0065-3527(08)60450-9 3914211

[B57] Stella S. Montoya G. (2016). The genome editing revolution: a CRISPR-Cas TALE off-target story. *Bioessays* 38 S4–S13. 10.1002/bies.201670903 27417121

[B58] Suenaga T. Kohyama M. Hirayasu K. Arase H. (2014). Engineering large viral DNA genomes using the CRISPR-Cas9 system. *Microbiol. Immunol.* 58 513–522. 10.1111/1348-0421.12180 25040500 PMC7168497

[B59] Sun N. Zhao H. (2013). Transcription activator-like effector nucleases (TALENs): a highly efficient and versatile tool for genome editing. *Biotechnol. Bioeng.* 110 1811–1821. 10.1002/bit.24890 23508559

[B60] Suweis S. Carr J. A. Maritan A. Rinaldo A. D’odorico P. (2015). Resilience and reactivity of global food security. *Proc. Natl. Acad. Sci. U.S.A.* 112 6902–6907. 10.1073/pnas.1507366112 25964361 PMC4460461

[B61] Takenaka K. Koshino-Kimura Y. Aoyama Y. Sera T. (2007). Inhibition of tomato yellow leaf curl virus replication by artificial zinc-finger proteins. *Nucleic Acids Symp. Ser. (Oxf.)* 429–430. 10.1093/nass/nrm215 18029770

[B62] Varsani A. Navas-Castillo J. Moriones E. Hernandez-Zepeda C. Idris A. Brown J. K. (2014). Establishment of three new genera in the family geminiviridae: becurtovirus, eragrovirus and turncurtovirus. *Arch. Virol.* 159 2193–2203. 10.1007/s00705-014-2050-2 24658781

[B63] Voytas D. F. Gao C. (2014). Precision genome engineering and agriculture: opportunities and regulatory challenges. *PLoS Biol.* 12:e1001877 10.1371/journal.pbio.1001877 PMC4051594 24915127

[B64] Waltz E. (2016a). CRISPR-edited crops free to enter market, skip regulation. *Nat. Biotechnol.* 34 582–582. 10.1038/nbt0616-582 27281401

[B65] Waltz E. (2016b). Gene-edited CRISPR mushroom escapes US regulation. *Nature* 532:293 10.1038/nature.2016.19754 27111611

[B66] Wang J. Quake S. R. (2014). RNA-guided endonuclease provides a therapeutic strategy to cure latent herpesviridae infection. *Proc. Natl. Acad. Sci. U.S.A.* 111 13157–13162. 10.1073/pnas.1410785111 25157128 PMC4246930

[B67] Whitfield A. E. Falk B. W. Rotenberg D. (2015). Insect vector-mediated transmission of plant viruses. *Virology* 47 278–289. 10.1016/j.virol.2015.03.026 25824478

[B68] Whitham S. A. Hajimorad M. R. (2016). “Plant genetic resistance to viruses,” in *Current Research Topics in Plant Virology*, eds Wang A. Zhou X. (Cham: Springer International Publishing), 87–111.

[B69] Woo J. W. Kim J. Kwon S. I. Corvalan C. Cho S. W. Kim H. (2015). DNA-free genome editing in plants with preassembled CRISPR-Cas9 ribonucleoproteins. *Nat. Biotechnol.* 33 1162–1164. 10.1038/nbt.3389 26479191

[B70] Wright A. V. Nunez J. K. Doudna J. A. (2016). Biology and applications of CRISPR systems: harnessing nature’s toolbox for genome engineering. *Cell* 164 29–44. 10.1016/j.cell.2015.12.035 26771484

[B71] Yuen K. S. Chan C. P. Wong N. H. Ho C. H. Ho T. H. Lei T. (2015). CRISPR/Cas9-mediated genome editing of Epstein-Barr virus in human cells. *J. Gen. Virol.* 96 626–636. 10.1099/jgv.0.000012 25502645

[B72] Zaidi S. S. Amin I. Iqbal Z. Akhtar K. P. Scheﬄer B. E. Mansoor S. (2016a). Sesbania bispinosa, a new host of a begomovirus-betasatellite complex in Pakistan. *Can. J. Plant Pathol.* 38 107–111. 10.1080/07060661.2015.1128980

[B73] Zaidi S. S. Mansoor S. Ali Z. Tashkandi M. Mahfouz M. M. (2016b). Engineering plants for geminivirus resistance with CRISPR/Cas9 system. *Trends Plant Sci.* 21 279–281. 10.1016/j.tplants.2016.01.023 26880316

[B74] Zaidi S. S. Martin D. P. Amin I. Farooq M. Mansoor S. (2016c). Tomato leaf curl New Delhi virus; a widespread bipartite begomovirus in the territory of monopartite begomoviruses. *Mol. Plant Pathol.* 10.1111/mpp.12481 [Epub ahead of print]. PMC6638225 27553982

[B75] Zaidi S. S. Shafiq M. Amin I. Scheﬄer B. E. Scheﬄer J. A. Briddon R. W. (2016d). Frequent occurrence of Tomato leaf curl New Delhi virus in cotton leaf curl disease affected cotton in Pakistan. *PLoS ONE* 11:e0155520 10.1371/journal.pone.0155520 PMC4877078 27213535

[B76] Zhang Y. Liang Z. Zong Y. Wang Y. Liu J. Chen K. (2016). Efficient and transgene-free genome editing in wheat through transient expression of CRISPR/Cas9 DNA or RNA. *Nat. Commun.* 7:12617 10.1038/ncomms12617 PMC5007326 27558837

[B77] Zhen S. Hua L. Liu Y. H. Gao L. C. Fu J. Wan D. Y. (2015). Harnessing the clustered regularly interspaced short palindromic repeat (CRISPR)/CRISPR-associated Cas9 system to disrupt the hepatitis B virus. *Gene Ther.* 22 404–412. 10.1038/gt.2015.2 25652100

[B78] Zhou X. P. (2013). Advances in understanding begomovirus satellites. *Annu. Rev. Phytopathol.* 51 357–381. 10.1146/annurev-phyto-082712-102234 23915133

